# The Relationship Between Biomarkers of Exercise-Induced Gastrointestinal Syndrome and Exercise-Associated Gastrointestinal Symptoms

**DOI:** 10.3390/sports14060248

**Published:** 2026-06-17

**Authors:** Keagan Hillemacher, Charlie Beaconsfield, Samuel Fitzgerald, Brooke Mahoney, Stephanie Gaskell, Rhiannon M. J. Snipe, Ricardo J. S. Costa

**Affiliations:** 1 Department of Nutrition, Dietetics & Food, Faculty of Medicine, Nursing and Health Sciences, Monash University, Level 1, 264 Ferntree Gully Road, Notting Hill, VIC 3168, Australia; keagan.hillemacher@monash.edu (K.H.); charliebeac11@gmail.com (C.B.); sfit0008@student.monash.edu (S.F.); bmah0013@student.monash.edu (B.M.); stephanie.gaskell@monash.edu (S.G.); 2 Centre for Sport Research, Institute for Physical Activity and Nutrition, School of Exercise and Nutrition Sciences, Faculty of Health, Deakin University, 221 Burwood Highway, Burwood, VIC 3125, Australia; r.snipe@deakin.edu.au

**Keywords:** exertional-heat stress, intestinal epithelium, permeability, bacterial endotoxin, systemic inflammation, ultra-endurance

## Abstract

Prolonged endurance exercise performed in hot ambient conditions is associated with an increased prevalence of performance-limiting gastrointestinal perturbations. This study aimed to examine the associations between biomarkers of exercise-induced gastrointestinal syndrome (EIGS) and exercise-associated gastrointestinal symptoms (Ex-GIS) under exertional heat stress (EHS). Fifty-six non-heat acclimated endurance-trained individuals completed 2 h of steady state running at 60% maximal oxygen uptake ($\dot{V}O_{2\max}$) with an ambient temperature of 35.1 °C and relative humidity 29.4%. Venous blood samples were collected immediately pre- and post-exercise to quantify plasma concentrations of gastrointestinal epithelial injury and systemic inflammatory biomarkers, whilst gastrointestinal symptoms were recorded at regular intervals throughout the exercise protocol. Spearman’s rank correlation identified moderately significant relationships between interleukin-6 (IL-6) with defecation-bloody stools; interleukin-10 (IL-10) with upper abdominal pain; and IL-10, IL-1 receptor antagonist (IL-1ra), and systemic inflammatory response (SIR) profile with flatulence. Simple linear regression demonstrated that IL-6 explained a small but significant proportion of the variance defecation-bloody stool (adjusted *R*^2^ = 0.094, *p* = 0.024); whilst variance in flatulence was independently explained by IL-10 (adjusted *R*^2^ = 0.138, *p* = 0.025), IL-1ra (adjusted *R*^2^ = 0.122, *p* = 0.033), and SIR-Profile (adjusted *R*^2^ = 0.112, *p* = 0.040). These findings suggest that Ex-GIS development is multifactorial in aetiology and pathophysiology, and that symptom reporting alone likely underestimates perturbations to the gastrointestinal tract during EHS.

## 1. Introduction

Exercise-induced gastrointestinal syndrome (EIGS) is a well-established phenomenon that limits exercise performance through disrupting gastrointestinal integrity and function, as well as prompting systemic immune responses [1]. Most commonly observed in endurance and ultra-endurance events, especially those with extreme environmental challenges (e.g., heat exposure), athletes frequently report exercise-associated gastrointestinal symptoms (Ex-GIS) as a major limiting factor to their performance [2,3]. The Ex-GIS experienced by athletes can range from mild discomfort to complete cessation of the exercise activity. These symptoms may originate from various areas along the gastrointestinal tract, including the upper GIS (i.e., gastro-oesophageal origin), lower GIS (i.e., intestinal origin), and other related symptoms (nausea, dizziness and acute transient abdominal pain (stitch)) [4]. Endurance exercise modalities of relatively short duration (e.g., 60 min), but moderate intensity (e.g., 70% maximal oxygen uptake ($\dot{V}O_{2\max}$)), are sufficient to induce intestinal epithelial cell injury and hyperpermeability, and subsequently translocation of intestinal luminal originating pathogens into systemic circulation that promote a systemic immune response [5,6]. Yet it appears that the incidence and severity of Ex-GIS increase in proportion to the duration and/or intensity of endurance exercise [7,8]. This has been observed in both field and laboratory settings, reported in approximately 45% of marathon runners [9]; and Ex-GIS has been reported to reach up to 96% in 161 km ultra-endurance events [10]. In addition to these exercise stressors, exertional-heat stress (EHS) is known to exacerbate perturbations in gastrointestinal epithelial integrity, increase pathogen translocation (i.e., bacteria and/or bacterial endotoxins), and promote systemic inflammation [11]. Although prolonged endurance exercise alone compromises gastrointestinal epithelial integrity, the current literature describes a threshold at which running for ≥2 h at 60% $\dot{V}O_{2\max}$ under EHS induces significant perturbations to gastrointestinal integrity and systemic responses, and promoted substantial Ex-GIS [12].

EIGS encompasses the physiological responses to exercise that disrupt gastrointestinal integrity and function, commonly leading to Ex-GIS. Two primary physiological pathways have been described in the pathophysiology of EIGS. Firstly, the circulatory-gastrointestinal pathway refers to splanchnic hypoperfusion and subsequent acute ischaemia; whereby, blood is redirected towards working skeletal muscles and peripheral circulation to aid muscle energy metabolism and thermoregulation, respectively [7,13,14,15]. Splanchnic hypoperfusion, together with elevated core body temperature, contributes to gastrointestinal barrier disruption through enterocyte damage, with intestinal fatty acid-binding protein (I-FABP) serving as a surrogate marker of this injury [16]. I-FABP is abundant in small intestinal enterocytes and is a sensitive marker of enterocyte damage, as it is rapidly released into circulation following cellular injury [16,17]. Enterocyte damage, as well as the opening of tight junction complexes, ultimately contributes to increased gastrointestinal tract permeability [17]. Lipopolysaccharide (LPS), derived from Gram-negative bacteria, may translocate across the epithelial barrier, and binds to lipopolysaccharide-binding protein (LBP) [18]. The LPS–LBP complex is presented to soluble CD14 (sCD14), activating downstream inflammatory signalling pathways, as evidenced by increased plasma levels of sCD14, immunoglobulin M (IgM), and nuclear factor-κB (NF-κB) [19,20,21]. Activation of NF-κB transcription factors upregulates the inflammatory cytokine cascade; including local and systemic increases in interleukin-1 beta (IL-1β), tumour necrosis factor-alpha (TNF-α), interleukin-6 (IL-6), and interleukin-10 (IL-10), exacerbating tight junction dysfunction and creating a positive feedback loop characterised by further increases in epithelial hyperpermeability and systemic inflammation [17,19,22].

Secondly, the neuroendocrine–gastrointestinal pathway: exercise-induced sympathetic nerve activation is synonymous with impaired gastrointestinal motility, secretion, absorption, and immune function [1,23,24]. This appears to result from dysregulated control by the myenteric and submucosal plexuses, as well as a reduced capacity of the interstitial cells of cajal to generate gastric slow-wave activity [25,26]. In a state of hypomotility, gastric emptying is delayed whilst orocaecal transit time increases [27,28,29]. The cumulative effect is nutrient malabsorption and increased luminal distension from fermentation of unabsorbed carbohydrates, thereby contributing to greater Ex-GIS [30]. Both anecdotal reports and the existing literature suggest that Ex-GIS is primarily associated with impaired functional capacity of the gastrointestinal tract during exercise, characterised by feeding intolerance and reduced motility [31,32]. Frequent reports of a high prevalence of Ex-GIS among athletes training or competing in endurance and ultra-endurance events, particularly in hot ambient conditions, have highlighted the need to identify preventative measures [1].

Research to date has predominantly focused on mapping the physiological pathways contributing to EIGS and identifying strategies to mitigate symptom incidence and severity [1]. Investigative studies utilise a range of biomarkers that reflect the key physiological mechanisms underpinning EIGS, including markers of gastrointestinal epithelial injury and systemic inflammation. There is no current data exploration that specifically examines the associations between perturbations to gastrointestinal integrity and systemic inflammatory markers with the incidence or severity of Ex-GIS under EHS. Rather, previous studies have primarily reported changes in circulating blood biomarkers without directly linking these responses to symptom outcomes. Thus, this study aimed to explore the relationships and associations among integrity markers of gastrointestinal epithelial damage, bacterial endotoxin translocation, systemic inflammatory responses, and reported Ex-GIS in trained endurance athletes under EHS. A secondary aim was to determine whether the variance in Ex-GIS could be explained by changes in individual biomarkers. It was hypothesised that biomarkers of epithelial cell damage and systemic inflammation would demonstrate significant associations with reported Ex-GIS, and that individual biomarker responses would explain a proportion of the variance in symptom outcomes.

## 2. Materials and Methods

### 2.1. Participants

A total of fifty-six non-heat acclimated endurance-trained runners [mean ± SD: (male *n* = 39, female *n* = 17) age: 34 ± 8 years, height 176 ± 9 cm, weight 71 ± 11 kg, % body fat 16 ± 6%, maximal oxygen uptake ($\dot{V}O_{2\max}$) 59 ± 8 mL/kg/min, weekly training volume 480 ± 280 min] were included. All participants provided written informed consent approved by Monash University Human Research Ethics Committee (MUHREC) and conformed to the Helsinki Declaration for Human Research Ethics [33]. All participants were pre-screened for injury and disease, including gastrointestinal infections and/or disorders (e.g., coeliac disease, inflammatory bowel disease, diverticular disease). Standard exclusion criteria was applied across all studies, this excluded participants consuming supplements considered as potential modifiers of gastrointestinal integrity (prebiotics, probiotics and/or antibiotics), following gastrointestinal dietary regimes (e.g., fermentable oligosaccharides, disaccharides, monosaccharides, and polyols (FODMAPs) and/or fibre modified diets) within the last 3 months, or consuming nonsteroidal anti-inflammatory medications and/or stool altering medications within 1 month prior to commencement of trials.

### 2.2. Research Design

**Figure 1:**
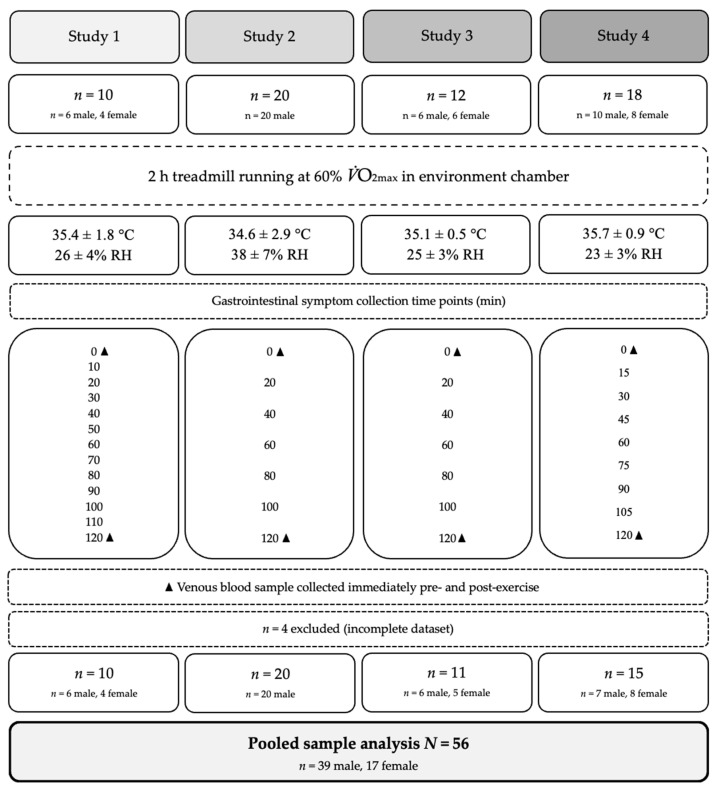
Schematic illustration of the pooled analysis procedure. Study 1 [34]; Study 2 [35]; Study 3 [36]; Study 4 [37]; $\dot{V}O_{2\max}$ = maximal oxygen uptake; °C = degrees Celsius; RH = relative humidity; min = minutes; ▲ = blood biomarker sampling timepoint; superscript numbers denote original study references.

Metadata from four independent previously published experimental studies were integrated in this exploratory pooled analysis [34,35,36,37]. The research approach taken is shown in Figure 1. The original pooled dataset comprised *N* = 60 participants across the four studies—four participants were excluded due to absent biomarker data. Studies were included if they employed a mirrored experimental design, and controlled EHS running protocol with concurrent assessment of gastrointestinal integrity biomarkers and Ex-GIS. Participants did not contribute data to more than one study; only data from the control arm of each study were extracted for the current analysis. Gastrointestinal symptom data were collected at different time intervals across the studies, including 10 min [34], 15 min [37], and 20 min intervals [35,36]. To ensure comparability across the pooled dataset, GIS scores were standardised prior to analysis to account for the difference in sampling frequency.

### 2.3. Preliminary Measures

Anthropometric measurements were obtained approximately one week prior to the main experimental trials, including height, nude body mass and body fat mass (Seca 515 MBCA, Seca Group, Hamburg, Germany). $\dot{V}O_{2\max}$ was determined using a continuous incremental exercise test to volitional exhaustion on a motorised treadmill using indirect calorimetry (Vmax Encore Metabolic Cart). To determine individual running speeds, the treadmill speed at approximately 60% $\dot{V}O_{2\max}$ and 1% gradient was extrapolated from the $\dot{V}O_{2}$-work rate relationship and then verified (10.2 ± 1.4 km/h).

### 2.4. Experimental Procedure

To minimise confounding effects of the lead-in diet on gastrointestinal responses, participants across all four studies were provided with a standardised 24 h low-FODMAP diet preceding each trial, with full dietary protocols reported in the original publication [34,35,36,37]. Meals were in accordance with best-practice guidelines for exercise gastroenterology and were individualised to meet each participant’s requirements [5]. Across the pooled sample, the mean 24 h lead-in low-FODMAP diet provided 10.9 MJ, comprising 406 g carbohydrate, 94 g protein, 57 g fat, and 43 g fibre, with a FODMAP content of less than 3.7 g. The standardised low-FODMAP breakfast provided 2.5 MJ, comprising 109 g carbohydrate, 18 g protein, 7 g fat, and 8 g fibre, with less than 1.5 g FODMAP, and was consumed with water at ~7:00 am.

All participants attended the laboratory at 8:00 am, approximately 1 h before the EHS trial commenced. Participants were instructed to void before a nude body mass assessment and to complete an exercise-specific gastrointestinal symptom assessment tool prior to exercise. Ex-GIS were assessed using a 10-point Likert-type rating scale (modified visual analogue scale (mVAS)) with established test–retest reliability [4,5]. Participants were educated and advised to complete a 0–10 rating scale as follows: 0 no GIS reported, 1–4 indicative of mild GIS (i.e., symptoms present but not sufficient to interfere with exercise workload), 5–9 indicative of severe GIS (i.e., symptoms substantial enough to interfere with exercise workload), and 10 indicative of extremely severe GIS (warranting exercise cessation). GIS were recorded at baseline and at regular intervals throughout the two-hour exercise protocol across all four studies. Reported GIS were categorised into upper GIS (i.e., belching, heartburn, upper abdominal pain, upper abdominal bloating, urge to regurgitate, regurgitation, projectile vomiting), lower GIS (i.e., lower abdominal pain, lower abdominal bloating, flatulence, urge to defecate, defecation-loose stools, diarrhoea, defecation-bloody stools) and other related symptoms (i.e., nausea, dizziness, and acute transient abdominal pain (stitch)). Rectal temperature (Tre) was continuously monitored during running using a thermocouple inserted 12–15 cm beyond the external anal sphincter (Alpha-Technics Precision Temperature 4600 Thermometer and Grant Squirrel data logger, Shepreth, UK). Participants completed 2 h (commencing at 9:00 am) of steady state treadmill running at 60% $\dot{V}O_{2\max}$ inside an environmental chamber. Ambient conditions were consistent across trials (Figure 1). Heart rate (HR), rating of perceived exertion (RPE) [38], Tre, thermal comfort rating [39], body mass and GIS were recorded throughout EHS. Water intake during exercise was permitted ad libitum [34,35,37], or controlled at (8 equal portions of 250 ± 40 mL consumed immediately before and every 15 min during exercise) [36]. Trials were conducted during cooler seasonal periods (temperatures consistently ≤20 °C) to minimise individual heat acclimatisation.

### 2.5. Biomarker Analysis

Venous blood samples were collected via venepuncture immediately pre- and post-exercise into heparin (6 mL, 1.5 IU/mL heparin) and EDTA vacutainers (4 mL, 1.6 mg/mL). Samples were centrifuged at 4000 rpm (1500 *g*) for 10 min within 15 min of collection. Plasma was aliquoted into micro storage tubes and stored at −80 °C until analysis. Plasma concentrations of I-FABP (HK406, Hycult Biotech, Uden, The Netherlands), soluble CD14 (sCD14; HK320, Hycult Biotech, Uden, The Netherlands), lipopolysaccharide binding protein (LBP; HK315, Hycult Biotech, Uden, The Netherlands), and endotoxin core antibody IgM (HK504, Hycult Biotech, Uden, The Netherlands) were quantified by ELISA in each individual study. Systemic inflammatory cytokines (TNF-α, IL-1β, IL-6, IL-8, IL-10, and IL-1ra) were measured via multiplex immunoassay using the HCYTOMAG-60K platform (EMD Millipore, Darmstadt, Germany) across [34,35,36] and the HCYTOMAG-28SK platform (MilliporeSigma, Darmstadt, Germany) in [37]. All variables were analysed in duplicate according to manufacturer instructions on the same day, with standards, controls, and each participant assayed on the same plate. An exercise-associated systemic inflammatory response (SIR) profile was derived by summing the peak pre- to post-exercise changes (Δ) in pro-inflammatory (IL-1β, TNF-α), response/modulatory (IL-6, IL-8), and anti-inflammatory (IL-10, IL-1ra) cytokines [5,35]. Absolute baseline cytokine concentrations (pg/mL) were subtracted from peak post-exercise values to quantify the magnitude of change, with summed values expressed as arbitrary units to represent overall systemic inflammation. The coefficient of variation (CV) ranged across studies and are reported in their original publication [34,35,36,37]. Not all biomarkers were measured across all four studies.

### 2.6. Statistical Analysis

Each individual experimental trial [34,35,36,37] included in this pooled analysis reported sufficient sample size to produce adequate statistical power. In addition, Post hoc power analysis was conducted using GPower 3.1 (Kiel, Germany) for correlation and simple linear regression analyses, assuming a medium effect size (r = 0.30; f^2^ = 0.15), α = 0.05 and β = 0.80. As not all biomarkers were measured across all four studies, and some participants had incomplete datasets, sample sizes vary across biomarker analyses and are reported accordingly throughout the results. Data within the text is presented as mean ± SD or mean and 95% confidence interval (CI). Each individual Ex-GIS is the cumulative symptom score for each participant, scores were standardised prior to pooling. Normality of biomarker data was assessed using the Kolmogorov–Smirnov test. As the data were not normally distributed, Spearman’s rank correlation coefficient was used to assess relationships between changes in biomarkers (Δ biomarker) and Ex-GIS. A Bonferroni correction (*p* < 0.0026) and Benjamini–Hochberg false discovery rate correction were applied post hoc to account for multiple comparisons, results are reported at the uncorrected *p* < 0.05 threshold. Correlation strength was determined to be very weak (<0.200), weak (0.200–0.399), moderate (0.400–0.599), and strong (≥0.600). Simple linear regression was performed for each individual biomarker and moderately associated symptom to determine if Δ biomarker could explain Ex-GIS outcomes. To meet the assumption of normality, biomarkers were log-transformed to produce a normal distribution. During transformation, each biomarker was shifted by adding a constant equal to the minimum value plus 1, ensuring all values were positive. Log-transformed data were checked for outliers; values exceeding three standard deviations from the mean were removed, resulting in the exclusion of one participant from the IL-6 model. An exploratory multiple regression analysis was conducted but not included in the results due to high intercorrelations among biomarkers (IL-10, IL-1ra, and SIR-Profile). Statistical analysis was completed using IBM SPSS Statistics version 30.0. (IBM Corp., Armonk, NY USA). Generative AI (CoPilot 2026. Microsoft, Redmond, WA, USA) was used to assist in the development of SPSS Syntax, all AI-assisted code was independently reviewed, tested, and validated by the authors to ensure analytical accuracy.

## 3. Results

Mean changes in plasma concentrations of biomarkers of intestinal injury, permeability, and systemic inflammation are presented in Table 1. No strong correlations were observed between biomarkers of EIGS and Ex-GIS. At baseline, 41% of participants (*n* = 23) reported at least one gastrointestinal symptom prior to exercise (upper abdominal bloating, 12.5%; urge to defecate, 12.5%; belching, 8.9%; dizziness, 8.9%; lower abdominal bloating, 8.9%; flatulence, 5.4%; upper abdominal pain, 5.4%; nausea, 3.6%; heartburn, 1.8%; urge to regurgitate, 1.8%; lower abdominal pain, 1.8%; defecation-lose stool, 1.8%; diarrhoea, 1.8%; stitch, 1.8%). Severity scores were generally mild (1–3 out of 10).

**Figure 2:**
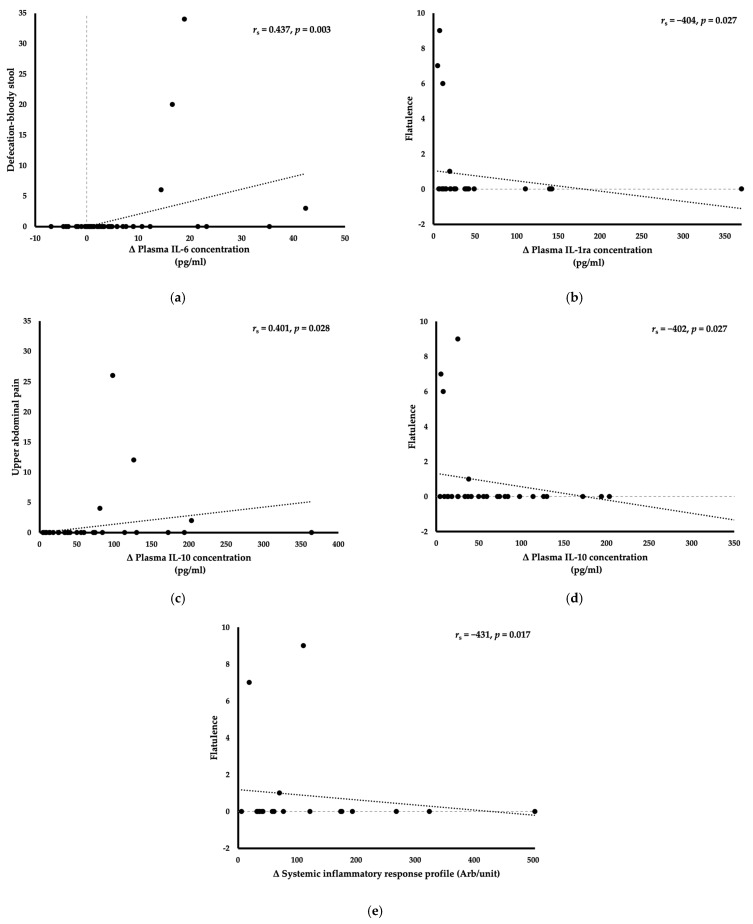
(**a**–**e**). Association between changes in plasma biomarker concentration and cumulative (summed) symptom score for exercise-associated gastrointestinal in response to 2 h of steady state running in hot ambient conditions (35.1 °C, 29.4% RH). IL-6 = interleukin-6; IL-10 = interleukin-10; IL-1ra = interleukin-1 receptor antagonist; SIR-Profile = systemic inflammatory response profile.

Correlation analyses assessing relationships between changes in EIGS biomarkers and Ex-GIS are presented in Table 2, with moderate significant correlations in Figure 2a–e. Biomarkers indicative of epithelial damage (I-FABP) and bacterial endotoxin translocation (LPB, sCD14 and IgM), demonstrated no moderate, statistically significant correlations (*r_s_* ≥ 0.4, *p* = 0.05) with any individual Ex-GIS. As shown in Table 2, IL-6 demonstrated a moderate positive correlation with defecation-bloody stools (*r_s_* = 0.437, *p* = 0.003). The anti-inflammatory cytokine IL-10 demonstrated a moderate positive correlation with upper abdominal pain (*r_s_* = 0.401, *p* = 0.028) and a moderate negative correlation with flatulence (*r_s_* = −0.402, *p* = 0.027). IL-1ra and SIR-Profile both showed a moderate negative correlation with flatulence (*r_s_* = −0.404, *p* = 0.027; *r_s_* = −0.431, *p* = 0.017, respectively).

Correlation analysis was repeated separately for male (*n* = 39) and female (*n* = 17) participants. Across the male subgroup, 13 significant correlations were identified. Upper abdominal pain demonstrated a moderate positive correlation with IL-6 (*r_s_* = 0.511, *p* = 0.002), and IL-10 (*r_s_* = 0.402, *p* = 0.042). Defecation of blood in stool showed a moderate positive correlation with TNF-α (*r_s_* = 0.416, *p* = 0.016), and IL-6 (*r_s_* = 0.429, *p* = 0.013). Flatulence demonstrated moderate negative correlations with IL1-b (*r_s_* = −0.407, *p* = 0.019), IL-10 (*r_s_* = −0.449, *p* = 0.021), IL-1ra (*r_s_* = −0.421, *p* = 0.032), and SIR-Profile (*r_s_* = −0.442, *p* = 0.024). Additionally, IL-10 demonstrated a moderate positive correlation with nausea (*r_s_* = 0.424, *p* = 0.031). Within the female subgroup, five significant correlations were identified, with notably stronger correlation coefficients than in the full cohort and male subgroup. I-FABP demonstrated moderate positive associations with both upper bloating (*r_s_* = 0.571, *p* = 0.017) and upper abdominal pain (*r_s_* = 0.571, *p* = 0.017). Nausea shared a strong positive correlation with TNF-α (*r_s_* = 0.671, *p* = 0.017) and a moderate positive correlation with IL-8 (*r_s_* = 0.592, *p* = 0.043). IgM demonstrated a moderate positive correlation with lower abdominal bloating (*r_s_* = 0.499, *p* = 0.049). Female subgroup sample sizes varied across biomarkers, ranging from *n* = 12 to *n* = 17; therefore, findings should be interpreted with considerable caution.

Table 3 presents the results of simple linear regression analyses conducted to determine whether pre- to post-exercise changes in individual biomarkers could explain variances in Ex-GIS outcomes. Changes in plasma IL-6 concentrations significantly explained 9.4% of the variance in defecation-bloody stools (F(1, 42) = 5.471, Adjusted R^2^ = 0.094, *p* = 0.024). IL-10 was the only biomarker that did not significantly explain variance in its corresponding symptom, with changes in plasma IL-10 concentrations not demonstrating a significant association with upper abdominal pain (F(1, 28) = 1.636, Adjusted R^2^ = 0.021, *p* = 0.211). Flatulence was significantly associated with by three independent biomarkers: IL-10 (F(1, 28) = 5.641, Adjusted *R*^2^ = 0.138, *p* = 0.025), IL-1ra (F(1, 28) = 5.011, Adjusted R^2^ = 0.122, *p* = 0.033), and SIR-Profile (F(1, 28) = 4.649, Adjusted R^2^ = 0.112, *p* = 0.04).

## 4. Discussion

The present pooled analysis examined the relationship between biomarkers of gastrointestinal epithelial injury, bacterial endotoxin translocation, systemic inflammatory responses, and Ex-GIS during EHS in trained endurance athletes. In accordance with the hypothesis, significant relationships were observed between plasma concentrations of I-FABP, IgM, IL-1β, IL-8, IL-10, IL-1ra, and the SIR-Profile with at least one individual Ex-GIS. Although the strength of many correlations was weak, only inflammatory cytokines IL-6, IL-10, IL-1ra, and the SIR-Profile demonstrated significant moderate correlations with Ex-GIS. Simple regression modelling found that IL-6 explained a small but significant proportion of the variance in defecation with bloody stool; whilst, IL-10, IL-1ra, and SIR-Profile each independently explained a modest proportion of the variance in flatulence. Findings suggest that the initial compromise in intestinal barrier function occurs largely asymptomatically; whereas, downstream inflammatory responses are more closely related to gastrointestinal symptoms. However, the modest explanatory power of individual biomarkers in regression analyses indicates that no single biomarker can reliably account for symptom variance, and that reliance on gastrointestinal symptom reporting alone likely underestimates the true perturbations of the gastrointestinal tract during EHS.

### 4.1. Epithelial Injury and Bacterial Translocation

Prolonged endurance exercise is known to impair gastrointestinal integrity by damaging the intestinal epithelial, resulting in the release of I-FABP from intestinal enterocytes [40,41]. Significant epithelial injury was confirmed, with plasma I-FABP concentration exceeding its minimum detectable change (MDC) of 1301 pg/mL [42], yet no meaningful associations were observed with any Ex-GIS. This suggests that whilst epithelial injury is likely integral to the development of EIGS, the degree of structural damage to the epithelial lining does not directly influence symptom expression. Following epithelial cell injury, adjunct hyperpermeability facilitates the translocation of luminal pathogens into circulation [43]. This increases endotoxin-handling capacity, as reflected by rising plasma LBP concentration and downstream pathways involving sCD14 and IgM [44]. Despite both markers increasing in response to the EHS, neither showed a significant association with Ex-GIS. An interesting finding was that despite the increase in plasma sCD14 concentration exceeding its MDC (780 ng/mL) [42], there was only a slight increase in plasma IgM concentration, consistent with previous literature [45]. The limited IgM response may reflect the capacity of trained endurance athletes who are known to have enhanced anti-endotoxin antibody responses and anti-inflammatory pathways [45]. Collectively, the observed elevations in biomarkers of epithelial cell injury and bacterial translocation in the absence of overt Ex-GIS suggest that athletes may experience clinically relevant gastrointestinal perturbations without subjective awareness. This has significant practical implications given the well-established role of endotoxin translocation in the pathogenesis of systemic inflammation. Reliance on symptom reporting alone will likely underestimate true bacterial endotoxin translocation, which, in extreme cases, has been implicated in septic shock and multi-organ failure [43,46], highlighting the clinical risk for athletes exercising in the heat.

### 4.2. Inflammatory Cytokine Response

The pre- to post-exercise changes in inflammatory cytokines observed in this study align with previous findings of comparable exercise protocols [32,47,48], demonstrating marginal shifts in pro-inflammatory cytokines (TNF-α and IL-1β), relative to greater responses in modulatory (IL-6 and IL-8) and anti-inflammatory cytokines (IL-10 and IL-1ra). Neither plasma concentrations changes in TNF-α nor IL-1β exceeded their respective MDC’s (14.6 pg/mL and 6.3 pg/mL). Indicating that whilst significant associations were observed with Ex-GIS, the magnitude of pro-inflammatory response was insufficient to elicit meaningful clinical changes despite established intestinal-barrier perturbations [42]. This limited pro-inflammatory response is consistent with the adaptive capacity of trained endurance athletes to attenuate acute inflammatory signalling during prolonged exercise [49].

IL-6 is a key mediator of both the exercise- and heat-induced inflammatory response [50]. The IL-6 response observed in the present study aligns with previous research demonstrating that while plasma IL-6 concentrations increase modestly during prolonged endurance exercise in temperate conditions, the addition of heat stress substantially amplifies this response [51,52]. Furthermore, 2 h of aerobic exercise found that heat exposure amplifies IL-6 response, with enhanced elevations at 30 °C and substantially greater magnitudes at 35 °C [34,53]. Increases in plasma IL-6 concentration explained a small but significant proportion of the variance in defecation with bloody stool. To our knowledge, no clear mechanistic link between IL-6 and defecation with bloody stools has been established in an exercise context. However, elevated plasma IL-6 concentrations have been associated with reduced colonic motility and gastrointestinal smooth muscle dysfunction [54,55].

The substantial increase in plasma IL-10 concentrations observed in this study appears to align with its well-established role as a counter-regulatory anti-inflammatory cytokine, activated in response to elevated plasma IL-6 concentrations [56]. Prolonged endurance exercise in temperate conditions has repeatedly increased circulating IL-10 concentrations, with exercise duration appearing to be a primary determinant of the magnitude of the response [57,58]. However, a marked increase in IL-10, compared with modest increases in pro-inflammatory cytokines, has been observed in trained individuals at core temperatures > 38.5 °C during walking under EHS (40 °C) [59]. The opposing associations of plasma IL-10 concentrations with upper abdominal pain and flatulence are the most novel findings of this analysis. The positive correlation between IL-10 and upper abdominal pain likely reflects an amplified inflammatory response compared with that typically observed under thermoneutral conditions [60]. Conversely, the negative correlation between IL-10 and flatulence may reflect its role in suppressing pro-inflammatory signalling, with animal models demonstrating that IL-10 deficiency directly impairs colonic contractility and motility [61]. By promoting greater epithelial integrity and motility, IL-10 may be linked with reduced intestinal dysfunction and, consequently, fewer gas-related symptoms. Given that IL-10 explained a modest proportion of the variance in flatulence but not in upper abdominal pain, its correlation with gastrointestinal symptoms may be more relevant to mechanisms involving intestinal barrier function and inflammatory regulation, rather than pain perception. Similarly, plasma IL-1ra concentrations mirrored IL-10 responses, demonstrating negative correlations with flatulence most likely due to overlapping upstream pathways [62]. The SIR-Profile, which integrates the cumulative changes in pro-, modulatory-, and anti-inflammatory cytokines, also demonstrated a moderate negative correlation with flatulence. Although IL-1ra and IL-10 underpin the SIR-Profile, all three independently explained variance in flatulence. The negative correlation between changes in plasma concentrations of IL-1ra, IL-10, and SIR-Profile with flatulence suggest that a stronger anti-inflammatory response may be linked to reduced gastrointestinal symptom severity roles in suppressing damage-generating pro-inflammatory processes [63]. Whilst these findings suggest that flatulence during EHS is related with changes in individual inflammatory biomarkers, the multicollinearity identified in exploratory multivariable analysis reflects the overlapping upstream signalling pathways shared between these markers.

The present pooled analysis examined associations between biomarkers of EIGS and Ex-GIS in endurance-trained athletes, underpinning the belief that symptom development has a multifactorial aetiology [12]. Ex-GIS demonstrated stronger correlations with systemic inflammatory markers than with markers of epithelial injury, suggesting that the preceding compromise in intestinal integrity may be asymptomatic. From a practical perspective, this implies that by the time Ex-GIS are reported, physiological perturbation is already established. This highlights the need for proactive physiological screening during endurance and ultra-endurance events, particularly in hot environmental conditions. It is therefore recommended that athletes adopt an individualised gastrointestinal management plan incorporating pre-race nutritional and hydration strategies to reduce modifiable risk factors, alongside routine physiological assessments at medical checkpoints during competition to support early detection of EIGS-related complications [1]. Inflammatory biomarker profiling may represent a beneficial screening tool, identifying athletes at heightened risk of EIGS development during EHS. This pooled analysis integrated data from four published studies over a six-year period, benefiting from consistent methodologies and controlled confounding factors [34,35,36,37], in line with best practice guidelines [5]. The two-hour EHS protocol was deliberately selected as an established experimental model to elicit the gastrointestinal physiological responses more commonly observed at exercise durations exceeding three hours [26].

### 4.3. Limitations

Several limitations of the present study exist, given that data has been extracted from four independent studies, which introduces inter-study variability. ELISA assays were performed independently across studies without lot-to-lot consistency. As noted, not all biomarkers were measured, resulting in variable sample sizes that reduced statistical power. The unequal sex distribution (male *n* = 39, female *n* = 17) further limits interpretability and prevents sex-stratified analysis. An important temporal limitation is that gastrointestinal symptom data were collected repeatedly throughout exercise, whilst biomarkers were only assessed at the beginning and end timepoints. Despite this exercise protocol eliciting significant change pre to post plasma concentrations of biomarkers LBP, TNF-α, and IL-1β did not surpass their MDC, potentially limiting the associations found between biomarkers and Ex-GIS as well as the modest predictability of biomarkers on symptom outcomes.

### 4.4. Future Research

Current research on the pathophysiological development of EIGS predominantly focuses on endurance-trained athletes [2]. Individuals also at risk of the negative outcomes of EIGS include those with cardiometabolic comorbidities, heat intolerance, presenting illness and/or infection and/or immunocompromised individuals. The underlying mechanistic pathways and Ex-GIS are also relevant to individuals in occupations involving prolonged physical exertion or EHS, such as military personnel, agricultural workers, miners, and firefighters. These populations, as well as novice athletes, are similarly at risk yet may lack the adaptive physiological responses observed in trained athletes. Future research should include occupational groups and untrained athletes to assess whether greater pathophysiological markers and symptom associations are present.

## 5. Conclusions

The present study found no strong correlations between biomarkers of EIGS and Ex-GIS in endurance-trained athletes during 2 h of steady-state running at 60% $\dot{V}O_{2\max}$ under EHS. Markers of epithelial injury and bacterial endotoxin translocation demonstrated weak- to no significant correlations with Ex-GIS. Whereas inflammatory cytokines (IL-6, IL-10, IL-1ra, and SIR-Profile) displayed only moderate significant correlations with individual Ex-GIS. Simple regression analysis showed that changes in plasma IL-6 concentration, explained a small but significant proportion of the variance in defecation with bloody stool, whilst changes in plasma concentrations of IL-10, IL-1ra, and the SIR-Profile each independently explained a moderate proportion of the variance in flatulence during exercise. Whilst inflammatory cytokine activation appears to contribute to the development of individual symptoms, the limited explanatory power of individual biomarkers indicates that symptom development is multifactorial in aetiology. Overall, these findings suggest that significant physiological perturbations of the gastrointestinal tract occur in the absence of overt symptoms during EHS. Practitioners and athletes should prioritise evidence-based preventative strategies during the pre-race and training period to reduce modifiable EIGS risk factors. Future research is required to explore the temporal relationship between biomarker change and symptom development.

## Figures and Tables

**Table 1:** Mean magnitude of change in gastrointestinal epithelial integrity and systemic inflammatory biomarkers of exercise-induced gastrointestinal syndrome in response to 2 h of steady state running in hot ambient conditions (35.1 °C, 29.4% RH).

		*N*	Mean Δ	95% CI		*Sig.*
				Lower Bound	Upper Bound	
I-FABP	pg/mL	56	1702	1324	2081	**<0.001**
LBP	ng/mL	35	1780	599	2961	0.171
sCD14	ng/mL	35	854	435	1273	**0.033**
IgM	MMU/mL	55	17.5	−0.5	35.4	**<0.001**
TNF-α	pg/mL	45	3.0	1.3	4.7	**<0.001**
IL-1β	pg/mL	45	1.3	0.2	2.3	**<0.001**
IL-6	pg/mL	45	6.0	3.1	8.9	**<0.001**
IL-8	pg/mL	45	5.5	2.5	8.4	**<0.001**
IL-10	pg/mL	30	73.8	44.6	102.9	**0.008**
IL-1ra	pg/mL	30	47.6	20.2	75.0	**<0.001**
SIR-Profile	arb/unit	30	145	93	197	**<0.001**

**Notes:** *n* = number of participants; Δ = mean change; CI = confidence interval; *Sig.* = significance level; statistical significance was set at *p* < 0.05, with significant results indicated in bold. I-FABP = intestinal fatty acid–binding protein; LBP = lipopolysaccharide-binding protein; sCD14 = soluble CD14; IgM = immunoglobulin M; TNF-α = tumour necrosis factor-alpha; IL-1β = interleukin-1 beta; IL-6 = interleukin-6; IL-8 = interleukin-8; IL-10 = interleukin-10; IL-1ra = interleukin-1 receptor antagonist; SIR-Profile = systemic inflammatory response profile.

**Table 2:** Correlations between exercise-associated gastrointestinal symptoms and plasma concentrations of biomarkers of gastrointestinal integrity and systemic inflammation during 2 h of steady state running in hot ambient conditions (35.1 °C, 29.4% RH).

		I-FABP	LBP	sCD14	IgM	TNF-α	IL-1β	IL-6	IL-8	IL-10	IL-1ra	SIR-Profile
	*N*	56	35	35	55	45	45	45	45	30	30	30
Power		0.751	0.570	0.570	0.745	0.667	0.667	0.667	0.667	0.514	0.514	0.514
Total GIS	*r_s_*	0.126	0.027	0.009	0.082	0.144	0.198	0.048	0.235	0.185	0.052	0.098
	*p*	0.355	0.879	0.958	0.552	0.344	0.191	0.757	0.120	0.327	0.785	0.606
Upper GIS	*r_s_*	0.089	0.023	−0.012	−0.033	0.005	0.041	−0.010	0.182	0.325	0.084	0.222
	*p*	0.516	0.894	0.945	0.812	0.975	0.787	0.947	0.230	0.079	0.660	0.238
Belching	*r_s_*	.	−0.106	−0.037	−0.03	−0.017	−0.059	−0.148	0.002	0.175	0.09	0.074
	*p*	.	0.544	0.834	0.829	0.910	0.699	0.332	0.988	0.356	0.637	0.697
Heartburn	*r_s_*	−0.097	−0.254	−0.205	0.008	0.214	0.192	0.240	**0.310**	0.068	−0.020	0.011
	*p*	0.475	0.141	0.237	0.953	0.158	0.206	0.112	**0.038**	0.720	0.916	0.953
Upper bloating	*r_s_*	0.190	0.044	0.122	−0.174	0.011	−0.073	−0.137	−0.002	0.027	−0.059	−0.097
	*p*	0.160	0.801	0.484	0.203	0.944	0.632	0.368	0.987	0.888	0.757	0.610
Upper abdominal pain	*r_s_*	0.085	−0.129	0.091	−0.137	0.235	**0.297**	0.273	0.279	**0.401**	0.299	0.311
	*p*	0.536	0.458	0.604	0.318	0.121	**0.048**	0.070	0.063	**0.028**	0.109	0.094
Urge to regurgitate	*r_s_*	0.033	−0.177	0.106	0.176	0.018	−0.041	0.01	0.156	0.097	0.097	0.05
	*p*	0.807	0.308	0.544	0.198	0.904	0.787	0.949	0.306	0.609	0.609	0.791
Regurgitation	*r_s_*	0.213	−0.136	−0.246	0.137	0.023	0.052	−0.035	0.139	.	.	.
	*p*	0.115	0.437	0.154	0.318	0.88	0.733	0.820	0.361	.	.	.
Vomit	*r_s_*	−0.155	.	.	0.245	0.209	0.180	0.197	0.023	0.204	0.182	0.161
	*p*	0.253	.	.	0.071	0.168	0.237	0.194	0.880	0.280	0.335	0.396
Lower GIS	*r_s_*	0.238	−0.063	−0.095	0.222	−0.065	−0.155	−0.149	0.007	−0.145	−0.221	−0.261
	*p*	0.077	0.718	0.587	0.104	0.672	0.308	0.328	0.963	0.444	0.242	0.164
Lower abdominal pain	*r_s_*	0.19	0.053	−0.054	−0.041	−0.083	−0.124	−0.005	−0.001	0.182	0.247	0.225
	*p*	0.16	0.761	0.760	0.767	0.589	0.417	0.972	0.995	0.335	0.189	0.231
Lower abdominal bloating	*r_s_*	−0.055	0.142	−0.147	0.208	−0.027	−0.017	−0.002	−0.094	−0.094	−0.107	−0.142
	*p*	0.686	0.415	0.400	0.127	0.859	0.910	0.988	0.537	0.622	0.573	0.454
Flatulence	*r_s_*	**0.286**	−0.065	0.036	**0.297**	−0.083	−0.27	−0.284	0.024	**−0.402**	**−0.404**	**−0.431**
	*p*	**0.033**	0.711	0.836	**0.028**	0.587	0.073	0.059	0.877	**0.027**	**0.027**	**0.017**
Urge to defecate	*r_s_*	0.175	0.067	−0.093	0.09	0.023	−0.01	−0.081	0.115	−0.226	−0.228	−0.246
	*p*	0.198	0.704	0.595	0.511	0.882	0.946	0.599	0.452	0.230	0.225	0.190
Defecation-loose stools	*r_s_*	0.213	−0.136	−0.246	0.137	0.023	0.052	−0.035	0.139	.	.	.
	*p*	0.115	0.437	0.154	0.318	0.880	0.733	0.820	0.361	.	.	.
Defecation-bloody stools	*r_s_*	−0.212	.	.	−0.082	**0.309**	0.251	**0.437**	0.221	0.336	0.201	0.267
	*p*	0.116	.	.	0.551	**0.039**	0.097	**0.003**	0.144	0.070	0.288	0.153
Nausea	*r_s_*	−0.099	−0.189	0.104	0.037	0.159	0.26	0.253	**0.315**	0.252	0.228	0.221
	*p*	0.467	0.277	0.553	0.787	0.296	0.085	0.094	**0.035**	0.179	0.226	0.241
Dizziness	*r_s_*	.	−0.202	0.199	0.049	**0.312**	0.204	0.181	0.288	0.219	0.022	0.049
	*p*	.	0.246	0.253	0.723	**0.037**	0.180	0.233	0.055	0.246	0.907	0.797
Stitch	*r_s_*	−0.134	−0.016	−0.096	−0.162	−0.165	0.089	−0.234	−0.171	−0.097	−0.225	−0.204
	*p*	0.326	0.925	0.585	0.238	0.279	0.561	0.122	0.261	0.612	0.231	0.280

**Notes:** *r_s_* = Spearman’s rank correlation coefficient; *p* = significance level. “.” indicates missing or non-calculable values. Statistical significance was set at *p* < 0.05, with significant results indicated in bold. Power was calculated post hoc using GPower 3.1 assuming a medium effect size r = 0.30 at α = 0.05. I-FABP = intestinal fatty acid–binding protein; LBP = lipopolysaccharide-binding protein; sCD14 = soluble CD14; IgM = immunoglobulin M; TNF-α = tumour necrosis factor-alpha; IL-1β = interleukin-1 beta; IL-6 = interleukin-6; IL-8 = interleukin-8; IL-10 = interleukin-10; IL-1ra = interleukin-1 receptor antagonist; SIR-Profile = systemic inflammatory response profile.

**Table 3:** Simple linear regression analysis predicting exercise-associated gastrointestinal symptoms from changes in plasma biomarkers of gastrointestinal integrity and systemic inflammation during 2 h of steady state running (35.1 °C, 29.4% RH).

Predictor	Symptom	*n*	*B*	*Std. Error B*	*β*	*F*	*R* ^2^	*Adjusted R* ^2^	*Sig.*
IL-6	Defecation-bloody stools	44	8.057	3.445	0.339	5.471	0.115	0.094	**0.024**
IL-10	Upper abdominal pain	30	2.532	1.98	0.235	1.636	0.055	0.021	0.211
IL-10	Flatulence	30	−1.937	0.816	−0.409	5.641	0.168	0.138	**0.025**
IL-1ra	Flatulence	30	−1.93	0.862	−0.390	5.011	0.152	0.122	**0.033**
SIR-Profile	Flatulence	30	−1.904	0.883	−0.377	4.649	0.142	0.112	**0.040**

**Notes:** *B* = unstandardized regression coefficient; *Std. Error B* = standard error of the coefficient; *F* = F-statistic; *R*^2^ = coefficient of determination; *Adjusted R*^2^ = adjusted coefficient of determination; *sig.* = significance level. Statistical significance was set at *p* < 0.05. Statistical power was 0.709 for *n* = 44 and 0.535 for models with *n* = 30. IL-6 = interleukin-6; IL-10 = interleukin-10; IL-1ra = interleukin-1 receptor antagonist; SIR-Profile = systemic inflammatory response profile.

## Data Availability

Data is unavailable due to privacy restrictions.

## Abbreviations

The following abbreviations are used in this manuscript:

EIGS	Exercise-induced gastrointestinal syndrome
Ex-GIS	Exercise-associated gastrointestinal symptoms
EHS	Exertional heat stress
I-FABP	Intestinal fatty acid binding protein
sCD14	Soluble CD14
IgM	Immunoglobulin M
LBP	Lipopolysaccharide binding protein
TNF-α	Tumour necrosis factor alpha
IL-1β	Interleukin-1 beta
IL-6	Interleukin-6
IL-8	Interleukin-8
IL-10	Interleukin-10
IL-1ra	Interleukin-1 receptor antagonist
SIR-Profile	Systemic inflammatory response Profile
